# Harm perceptions of electronic cigarettes and nicotine: A nationally representative cross-sectional survey of young people in Great Britain

**DOI:** 10.1016/j.drugalcdep.2018.08.016

**Published:** 2018-11-01

**Authors:** Katherine East, Leonie S. Brose, Ann McNeill, Hazel Cheeseman, Deborah Arnott, Sara C. Hitchman

**Affiliations:** a National Addiction Centre, Institute of Psychiatry, Psychology and Neuroscience, King’s College London, 4 Windsor Walk, Denmark Hill, SE5 8BB, London, UK; b UK Centre for Tobacco and Alcohol Studies, University of Nottingham, NG5 1PB, Nottingham, UK; c Action on Smoking and Health UK, 6th floor, Suites 59-63, New House, 67-68 Hatton Garden, EC1N 8JY, London, UK

**Keywords:** Electronic cigarette, Smoking, Tobacco, Nicotine, Harm perception, Youth

## Abstract

**Background:**

E-cigarettes often contain nicotine without the most harmful constituents of tobacco smoke.

**Aims:**

This study aims to assess prevalence and correlates of accurately perceiving e-cigarettes as less harmful than cigarettes and that none or a small amount of the harm from smoking comes from nicotine.

**Methods:**

Cross-sectional survey of 2,103 11–18-year-olds in Great Britain in 2016. Prevalence of e-cigarette and nicotine harm perceptions were calculated. Logistic regressions assessed associations between accurate e-cigarette and nicotine harm perceptions and smoking, e-cigarette use, gender, age, region, social grade, family smoking, family e-cigarette use, smoking friends, public approval of smoking, and public approval of e-cigarettes. Associations between accurate e-cigarette and nicotine harm perceptions were also assessed.

**Results:**

Most (63.4%) accurate e-cigarette harm perceptions were higher among those aged 16+ (OR = 1.89 [95%CI = 1.45–2.47]), 14–15 (OR = 1.29 [1.00–1.65]), who tried/used an e-cigarette sometimes (OR = 1.51 [1.03–2.21]), with family e-cigarette use (OR = 2.11 [1.46–3.04]), who perceived public disapproval of smoking (OR = 2.11 [1.18–3.77]) and approval of e-cigarettes (OR = 2.44 [1.73–3.45]), and with accurate nicotine harm perceptions (OR = 2.05 [1.28–3.28]). Accurate nicotine harm perceptions were higher among those aged 16+ (OR = 2.60 [1.62–4.16]), from North England (OR = 1.87 [1.02–3.43]) and Wales/Scotland (OR = 2.61 [1.35–5.03]) vs. London, with family smoking (OR = 1.59 [1.05–2.42]), and with accurate e-cigarette harm perceptions (OR = 2.12 [1.32–3.41]).

**Conclusions:**

Many young people have inaccurate harm perceptions of e-cigarettes and nicotine. Accurate e-cigarette and nicotine harm perceptions were associated with one another. E-cigarette use was associated with accurate e-cigarette but not nicotine harm perceptions; smoking was not associated with either.

## Highlights

• Two thirds (63%) had accurate harm perceptions of e-cigarettes.
• Few (9%) had accurate harm perceptions of nicotine.
• Accurate harm perceptions of e-cigarettes and nicotine were positively associated.
• E-cigarette users, but not smokers, had more accurate e-cigarette harm perceptions.
• Neither smoking nor e-cigarette use were associated with nicotine harm perceptions.

## 1 Introduction

Tobacco smoking is the leading cause of preventable death and disability worldwide (WHO, 2005, 2015). While it is primarily the nicotine in cigarettes that is addictive, most of the health harms of smoking are caused by other constituents of tobacco smoke (Benowitz, 2009). Electronic (e-)cigarettes are battery-powered devices that heat a solution usually containing nicotine, flavourings, propylene glycol and/or vegetable glycerin to produce an inhalable aerosol. As e-cigarettes do not contain tobacco and do not involve combustion, current evidence suggests they are less harmful to both users and others relative to tobacco cigarettes (Czogala et al., 2014; Hajek et al., 2014; Harrell et al., 2014; McAuley et al., 2012; McNeill et al., 2015, 2018; Tobacco Advisory Group of the Royal College of Physicians, 2016).

Over the past decade, there have been many studies assessing the relative harm perceptions of e-cigarettes compared to cigarettes. Depending on the sample, year of data collection, and country, the proportion who accurately perceive that e-cigarettes are less harmful than cigarettes varies between 17% and 82% (Adkison et al., 2013; Ambrose et al., 2014; Amrock et al., 2015; Anand et al., 2015; Brose et al., 2015; Eastwood et al., 2017; Majeed et al., 2017; McNeill et al., 2018; Nayak et al., 2017; Persoskie et al., 2017; Richardson et al., 2014; Thrasher et al., 2016; Yong et al., 2017). In adults from the U.S. (Majeed et al., 2017) and Great Britain (Brose et al., 2015; McNeill et al., 2018), it appears that e-cigarettes are being perceived as increasingly harmful over time. Similar patterns are seen among young people in Great Britain, and the proportion who accurately perceive e-cigarettes as less harmful than smoking decreased from 73% in 2013 through 67% in 2014 and 2015 to 62% in 2016 (Eastwood et al., 2017).

Harm perceptions of nicotine are often also incorrect. Compared with tobacco, the harms of sustained nicotine use are negligible, and there is little evidence that nicotine increases cancer risk (Tobacco Advisory Group of the Royal College of Physicians, 2016). However, between 2002 and 2008, only 40–50% of adults in the UK, the US, Australia, and Canada accurately perceived that nicotine is not the chemical that causes most of the cancer in cigarettes (Borland et al., 2011), and this has not changed dramatically since (McNeill et al., 2018). In England in 2009, 40% of adult smokers perceived that long-term use (a year or more) of nicotine replacement therapy (NRT) was not harmful, and of the 31% who perceived that long-term NRT use was harmful, the most commonly reported concerns were addiction, lung cancer, and mouth cancer (Black et al., 2012). Among daily adult smokers from Sweden and Norway, 15% perceived that none or a very small part of the health risks from smoking come from nicotine, while 31% perceived a relatively small part (Wikmans and Ramström, 2010). To-date, there has been little research on the harm perceptions of nicotine among young people, but one study of U.S. Freshmen in 2004 found that 80% accurately reported the nicotine patch to be less harmful than smoking, with corresponding proportions of 76% for nicotine gum and 47% for the nicotine inhaler (Smith et al., 2007). Therefore, the perceived harm of nicotine may depend on the product or the route of administration.

The proportion of inaccurate harm perceptions of e-cigarettes and nicotine is concerning, as it may limit the proportion of smokers willing to try or use a less harmful product which may help them quit. Indeed, adult smokers and ex-smokers who accurately perceive e-cigarettes as less harmful than smoking are more likely to try or currently use an e-cigarette than those who do not hold this perception (Adkison et al., 2013; Brose et al., 2015; Yong et al., 2017). However, this same association has been found among both smoking and non-smoking young people (Ambrose et al., 2014; Amrock et al., 2015; Persoskie et al., 2017; Thrasher et al., 2016), which could be problematic if accurate harm perceptions are encouraging never-smokers to try or use e-cigarettes. On the other hand, young people who perceive that e-cigarettes and smoking are equally as harmful or that e-cigarettes are more harmful may be more likely to transition from e-cigarette use to smoking than those who perceive e-cigarettes as less harmful than smoking. However, there is no research on this to date.

Evidence for associations between harm perceptions of nicotine (or NRT) and using nicotine products is mixed. Borland et al. (2011) found an association between perceiving NRT as less harmful than smoking and using NRT in the past year among smokers and ex-smokers, while Black et al. (2012) found no such association.

In addition to assessing associations between accurate harm perceptions and use of both tobacco cigarettes and e-cigarettes, it is also important to identify potential demographic and social correlates of accurate harm perceptions. It is plausible that exposure to smoking and e-cigarettes by family and friends and perceived approval of these products will influence harm perceptions, particularly among youth, whose smoking and e-cigarette uptake has been found to be influenced by some of these social factors (Chang et al., 2006; East et al., 2018; Forrester et al., 2007; Leonardi-Bee et al., 2011; O’Loughlin et al., 2009, 2014).

To our knowledge, there has been no published data on associations between harm perceptions of e-cigarettes and harm perceptions of nicotine among young people. As e-cigarettes often, although not always, contain nicotine, it should be expected that harm perceptions of both would be correlated.

Given this, this study has the following two aims: (1) to assess the prevalence of harm perceptions of (a) e-cigarettes relative to cigarettes and (b) nicotine in relation to smoking among young people in Great Britain and (2) to assess the correlates of accurate perceptions that (a) e-cigarettes are less harmful than cigarettes and (b) none or a small amount of the harm from smoking comes from nicotine among young people in Great Britain.

## 2 Methods

### 2.1 Design and procedure

Data were drawn from the cross-sectional 2016 Action on Smoking and Health (ASH) Smokefree Great Britain Youth survey conducted between 11th March and 10th April 2016. This survey is commissioned annually by ASH and is drawn from an online panel maintained by YouGov PLC. Full details of YouGov PLC’s online panel are described in Eastwood et al. (2015). Respondents age 16–18 were sampled directly from YouGov PLC’s online panel via an email informing them of the survey and inviting them to take part. Respondents age 11–15 were recruited via emails to parents or legal guardians from the YouGov panel and asked them to read the information about the survey and pass it on to their child if they and their child consented to participation. YouGov adheres to the code of conduct set out by the Market Research Society (https://www.mrs.org.uk/pdf/mrs%20code%20of%20conduct%202014.pdf). Respondents consent to completing surveys in return for a modest financial incentive (50p for those aged 16–18, £1.50 for those aged 11–15); respondents age 11–15 also required consent from their parents or legal guardians.

### 2.2 Sample

The sample was designed to be nationally representative in terms of age, gender, and region. The survey was completed by 2,331 respondents age 11–18 inclusive. Respondents who had never heard of e-cigarettes (n = 159), did not report smoking status (n = 32) or e-cigarette status (n = 6), or had missing data for social grade (n = 54) were excluded from the analyses (9.8%). This left 2103 respondents (90.2%) in the study sample.

### 2.3 Measures

All measures included in this study, item wording, response options, and coding are listed below.

#### 2.3.1 Harm perceptions

*Relative harm perception of e-cigarettes*. Participants were asked: “Compared to cigarettes, do you think e-cigarettes are more or less harmful to the person using them, or is there no difference?” (a) “Less harmful”, (b) “About the same”, (c) “More harmful”, or (d) “Don’t know”. For analysis of correlates of accurate harm perceptions, responses were dichotomised as less harmful (accurate perception) (a) vs. otherwise (b–d).

*Harm perception of nicotine*. Participants were asked: “According to what you know or believe, how much of the harm from smoking cigarettes comes from nicotine?” (a) “None or very small”, (b) “Some but well under half the risk”, (c) “Around half the risk”, (d) “Much more than half the risk”, (e) “Nearly all the risk”, or (f) “Don’t know”. For analysis of correlates of accurate harm perceptions, responses were dichotomized as none or very small (accurate perception) (a) vs. otherwise (b–f). This measure was adapted from Wikmans and Ramström (2010).

#### 2.3.2 Smoking and e-cigarette status

*Smoking status*. Participants identified “Which ONE of the following BEST applies to you?”: (a) “I have never smoked cigarettes, not even a puff or two”, (b) “I have only ever tried smoking cigarettes once”, (c) “I used to smoke sometimes but I never smoke cigarettes now” (d) “I sometimes smoke cigarettes now but less than one a week”, (e) “I usually smoke between one and six cigarettes a week”, (f) “I usually smoke more than six cigarettes a week”, or (g) “Don’t want to say”. For analysis, responses were coded as never (a), tried (b), ex-smoker (c), sometimes (d) and at least weekly (e–f); (g) was excluded.

*E-cigarette status*. Participants identified “Which ONE of the following is closest to describing your experience of e-cigarettes?” (a) “I have never used an e-cigarette”, (b) “I have only tried an e-cigarette once or twice”, (c) “I use e-cigarettes sometimes, but no more than once a month”, (d) “I use e-cigarettes more than once a month, but less than once a week”, (e) “I use e-cigarettes more than once a week but not every day”, (f) “I use e-cigarettes every day”, or (g) “Don’t want to say”. For analysis, responses were coded as never (a), tried or use sometimes (b–c), and at least monthly (d–f); (g) was excluded.

#### 2.3.3 Social norms

*Family smoking*. Participants were asked “Who in your family, if anyone, smokes tobacco cigarettes at the moment? Please tick all that apply” (a) Mother (or female carer)”, (b) “Father (or male carer)”, or (c) “Brother or sister”. For analysis, respondents were coded as having at least one family member who smokes if they selected any of (a) through (c).

*Family e-cigarette use*. Participants were asked “And who in your family, if anyone, uses e-cigarettes at the moment? Please tick all that apply” (a) Mother (or female carer)”, (b) “Father (or male carer)”, or (c) “Brother or sister”. For analysis, respondents were coded as having at least one family member who uses an e-cigarette if they selected any of (a) through (c).

*Number of smoking friends*. Participants were asked “Please think of the three friends you spend most time with. How many of them smoke tobacco cigarettes on a regular basis?” “0 (none of them)” “1”, “2”, “3 (all of them)”, “Don’t know”, or “Don’t want to say”. For analysis, responses were coded as “0 (none of them)”, “1”, “2”, “3 (all of them)” and don’t know/refused (“Don’t know”, “Don’t want to say”).

*Perceived public approval of smoking*. Participants were asked “In your opinion, do people in general approve or disapprove of… People smoking tobacco cigarettes?” (a) “Strongly approve”, (b) “Approve, (c) Neither approve nor disapprove, (d) “Disapprove”, (e) “Strongly disapprove”, or (f) “Don’t know”. For analysis, responses were coded as approve (a–b), neither (c), disapprove (d–e), and don’t know (f).

*Perceived public approval of e-cigarettes*. Participants were asked: “In your opinion, do people in general approve or disapprove of… People using e-cigarettes or vaping devices?” (a) “Strongly approve”, (b) “Approve, (c) Neither approve nor disapprove, (d) “Disapprove”, (e) “Strongly disapprove”, or (f) “Don’t know”. For analysis, responses were coded as approve (a–b), neither (c), disapprove (d–e), and don’t know (f).

#### 2.3.4 Demographics

Gender was recorded as male vs. female. Age was coded as 11–13 vs. 14–15 vs. 16–18. Participants’ region in Great Britain was coded as North England (North East, North West, Yorkshire, East Midlands, West Midlands) vs. South England (East, South East, South West) vs. London vs. Wales/Scotland. Social grade was coded as ABC1 (higher and intermediate managerial, administrative, supervisory, clerical and junior managerial, administrative, professional occupations) vs. C2DE (skilled, semi-skilled and unskilled manual occupations, unemployed and lowest grade occupations). Social grade was based on the occupation of the chief income earner in the household and was asked of the parents of those respondents age 11–15 and directly of those respondents age 16–18.

### 2.4 Statistical analysis

All data were weighted to be representative of age, gender, and region using the 2015 Office for National Statistics census data. All data reported, including sample characteristics, are weighted unless otherwise stated. All analyses used STATA 15.0 (StataCorp, 2017).

To assess the prevalence of harm perceptions of e-cigarettes relative to cigarettes (aim 1a) and nicotine (aim 1b), prevalence estimates and 95% confidence intervals were calculated.

To assess correlates of accurate perceptions that e-cigarettes are less harmful than cigarettes (aim 2a), unadjusted and adjusted logistic regression analyses were used to explore the association between accurately perceiving e-cigarettes to be less harmful than cigarettes (vs. otherwise) and smoking status, e-cigarette status, demographics (gender, age, region, social grade), social norms (family smoking, family e-cigarette use, number of smoking friends, perceived public approval of smoking, perceived public approval of e-cigarettes), and accurately perceiving that none or a very small amount of the harm from smoking comes from nicotine.

To assess correlates of accurate perceptions that none or a small amount of the harm from smoking comes from nicotine (aim 2b), unadjusted and adjusted logistic regression analyses were used to explore the association between accurately perceiving none or a very small amount of harm from smoking cigarettes comes from nicotine (vs. otherwise) and smoking status, e-cigarette status, demographics and social norms (as stated previously), and accurately perceiving that e-cigarettes are less harmful than smoking.

## 3 Results

### 3.1 Sample characteristics

Approximately half of respondents were male (51.5%), the majority were from higher social grades ABC1 (68%), and 35.2% were aged 11–13 years, 25.6% aged 14–15 years, and aged 39.2% 16–18 years. Just under half (41.9%) were from North England, 33.5% from South England, 10.9% from London, and 13.8% from Wales/Scotland. The majority were never smokers (80.7%) and never e-cigarette users (87.1%).

### 3.2 Aim 1a: Prevalence of harm perceptions of e-cigarettes relative to cigarettes

Almost two thirds (n = 1,331, 63.4%, 95% CI = 61.1–65.6) of respondents accurately perceived that e-cigarettes were less harmful than cigarettes, while 488 (22.9%, 95% CI = 21.0–24.9) perceived they were equally as harmful, 56 (2.6%, 95% CI = 2.0–3.4) perceived they were more harmful, and 228 (11.2%, 95% CI = 9.8–12.7) didn’t know.

### 3.3 Aim 1b: Prevalence of harm perceptions of nicotine

Only a small proportion (n = 183, 8.6%, 95% CI = 7.4–10.1) of respondents accurately perceived that none or a very small amount of the harm from smoking comes from nicotine, while corresponding values were 385 (17.7%, 95% CI = 15.9–19.6) for “some but well under half of the harm”, 420 (20.0%, 95% CI = 18.1–21.9) for “half of the harm”, 377 (17.9%, 95% CI = 16.2–19.7) for “much more than half of the harm”, 408 (20.2%, 95% CI = 18.4–22.0) for “nearly all of the harm”, and 330 (15.6%, 95% CI = 14.1–17.4) for “don’t know”.

### 3.4 Aim 2a: Correlates of the accurate perception that e-cigarettes are less harmful than cigarettes

In both adjusted and unadjusted analyses, respondents had higher odds of accurately perceiving that e-cigarettes are less harmful than cigarettes if they tried or sometimes use e-cigarettes, were older, had at least one family member who used e-cigarettes, had no smoking friends (vs. responding don’t know or refusing to say), perceive that the public approve or neither approves nor disapproves of e-cigarettes, and accurately perceive that none or a very small amount of the harm from smoking comes from nicotine (Table 1). In adjusted analyses only, respondents had higher odds of accurately perceiving that e-cigarettes are less harmful than cigarettes if they perceive that the public disapproves of smoking (Table 1). Smoking status and having at least one family member who smokes were not associated with accurate relative harm perceptions of e-cigarettes in adjusted analyses (Table 1).

**Table 1:** Adjusted and unadjusted logistic regression analyses of the accurate perception that e-cigarettes are less harmful than cigarettes (vs. otherwise), N = 2,103.

	N^1^ (% total sample)	E-cigarettes are less harmful than cigarettes (vs. otherwise)				
			Unadjusted		Adjusted^2^	
		%^1^	OR (95% CI)	p	OR (95% CI)	P
Smoking status						
Never (ref)	1634 (80.71)	61.53	1.00		1.00	
Tried	246 (10.25)	69.44	**1.42 (1.04-1.95)**	**.030**	1.00 (0.69-1.46)	.981
Ex-smoker	75 (3.57)	71.53	1.57 (0.80-3.10)	.193	1.04 (0.48-2.29)	.915
Sometimes	75 (2.67)	74.27	**1.80 (1.00-3.26)**	**.050**	1.06 (0.54-2.08)	.859
At least weekly	73 (2.81)	72.98	1.69 (0.95-3.00)	.075	1.47 (0.60-3.62)	.401

E-cigarette status						
Never (ref)	1796 (87.10)	61.41	1.00		1.00	
Tried/use sometimes	274 (11.28)	78.19	**2.25 (1.63-3.12)**	**<.001**	**1.51 (1.03-2.21)**	**.035**
At least monthly	33 (1.62)	64.72	1.15 (0.52-2.56)	.727	0.67 (0.23-2.01)	.479

Gender						
Male (ref)	966 (51.45)	65.44	1.00		1.00	
Female	1137 (48.55)	61.16	0.83 (0.69-1.01)	.061	0.86 (0.70-1.05)	.143

Age						
11-13 (ref)	698 (35.18)	55.09	1.00		1.00	
14-15	487 (25.59)	62.04	**1.33 (1.05-1.69)**	**.018**	**1.29 (1.00-1.65)**	**.049**
16-18	918 (39.22)	71.63	**2.06 (1.63-2.59)**	**<.001**	**1.89 (1.45-2.47)**	**<.001**

Region						
London (ref)	256 (10.85)	62.71	1.00		1.00	
North England	812 (41.85)	62.82	1.00 (0.73-1.38)	.976	1.10 (0.78-1.56)	.576
South England	732 (33.48)	64.82	1.10 (0.79-1.52)	.580	1.04 (0.81-1.62)	.445
Wales/Scotland	303 (13.82)	61.96	0.97 (0.67-1.41)	.867	0.98 (0.66-1.45)	.901

Social grade						
ABC1 (ref)	1432 (68.34)	63.65	1.00		1.00	
C2DE	671 (31.66)	62.73	1.42 (1.04-1.95)	.703	0.95 (0.77-1.18)	.646

Family smoking						
None (ref)	1576 (75.44)	61.74	1.00		1.00	
≥ 1 member	527 (24.56)	68.32	**1.34 (1.07-1.67)**	**.012**	1.05 (0.81-1.35)	.720

Family e-cigarette use						
None (ref)	1843 (87.46)	61.25	1.00		1.00	
≥ 1 member	260 (12.54)	78.03	**2.25 (1.62-3.12)**	**<.001**	**2.11 (1.46-3.04)**	**<.001**

Number smoking friends						
None (ref)	1629 (78.96)	63.48	1.00		1.00	
1	246 (10.45)	67.24	1.18 (0.87-1.61)	.294	0.86 (0.60-1.22)	.382
2	89 (4.11)	72.94	1.55 (0.91-2.64)	.106	0.97 (0.51-1.87)	.930
3	63 (2.57)	64.57	1.05 (0.58-1.88)	.873	0.52 (0.23-1.15)	.106
DK/Refused	76 (3.90)	39.58	**0.38 (0.23-0.61)**	**<.001**	**0.44 (0.25-0.77)**	**.004**

Public approval of smoking						
Approve (ref)	91 (3.89)	62.18	1.00		1.00	
Disapprove	1565 (75.47)	65.10	1.13 (0.71-1.81)	.596	**2.11 (1.18-3.77)**	**.011**
Neither	360 (16.34)	62.27	1.00 (0.60-1.67)	.988	1.20 (0.65-2.20)	.556
DK	87 (4.30)	37.99	**0.37 (0.20-0.71)**	**.003**	1.38 (0.57-3.37)	.477

Public approval of e-cigarettes						
Disapprove (ref)	800 (38.46)	56.33	1.00		1.00	
Approve	347 (15.95)	75.03	**2.33 (1.72-3.16)**	**<.001**	**2.44 (1.73-3.45)**	**<.001**
Neither	816 (38.59)	69.55	**1.77 (1.42-2.21)**	**<.001**	**2.01 (1.55-2.59)**	**<.001**
DK	140 (6.99)	41.18	**0.54 (0.37-0.80)**	**.002**	0.87 (0.50-1.50)	.617

How much harm from smoking comes from nicotine?						
Otherwise (ref)	1,920 (91.35)	61.69	1.00		1.00	
None or a very small amount	183 (8.65)	80.99	**2.65 (1.69- 4.14)**	**<.001**	**2.05 (1.28-3.28)**	**.003**
Constant					**0.42 (0.22-0.82)**	**.011**

DK = Don’t Know. ^1^N are unweighted, % are weighted. ^2^Analyses are adjusted for all variables listed.

### 3.5 Aim 2b: Correlates of the accurate perception that none or a very small amount of the harm from smoking comes from nicotine

In both adjusted and unadjusted analyses, respondents had higher odds of accurately perceiving that none or a very small amount of the harm from smoking comes from nicotine if they were older, from Wales/Scotland compared to London, had at least one family member who smokes, and accurately perceived that e-cigarettes are less harmful than smoking (Table 2). In adjusted analyses only, respondents had higher odds of accurately perceiving that none or a very small amount of the harm from smoking comes from nicotine if they were from the North of England compared with London but lower odds if they responded “don’t know” to perceived public approval of e-cigarettes (Table 2). Gender, smoking, and e-cigarette status were not associated with harm perceptions of nicotine in adjusted analyses (Table 2).

**Table 2:** Adjusted and unadjusted logistic regression analyses of the accurate perception that none or a very small amount of the harm from smoking comes from nicotine (vs. otherwise), N = 2,103.

	None or a very small amount of the harm from smoking comes from nicotine (vs. otherwise)				
		Unadjusted		Adjusted^2^	
	%^1^	OR (95% CI)	p	OR (95% CI)	p
Smoking status					
Never (ref)	7.47	1.00		1.00	
Tried	12.72	**1.81 (1.10-2.97)**	**.019**	1.10 (0.60-2.02)	.758
Ex-smoker	13.05	1.86 (0.77-4.49)	.167	1.01 (0.33-3.07)	.982
Sometimes	11.48	1.61 (0.70-3.69)	.262	0.74 (0.30-1.81)	.507
At least weekly	19.42	**2.99 (1.58-5.66)**	**.001**	2.25 (0.87-5.83)	.093

E-cigarette status					
Never (ref)	7.56	1.00		1.00	
Tried/use sometimes	16.78	**2.47 (1.66-3.66)**	**<.001**	1.57 (0.92-2.68)	.100
At least monthly	10.64	1.46 (0.49-4.29)	.495	0.62 (0.18-2.18)	.457

Gender					
Male (ref)	10.07	1.00		1.00	
Female	7.14	**0.69 (0.49-0.96)**	**.029**	0.72 (0.51-1.02)	.067

Age					
11-13 (ref)	4.82	1.00		1.00	
14-15	7.62	**1.63 (1.00-2.65)**	**.049**	1.52 (0.92-2.48)	.099
16-18	12.74	**4.58 (3.50-6.00)**	**<.001**	**2.60 (1.62-4.16)**	**<.001**

Region					
London (ref)	5.95	1.00		1.00	
North England	8.50	1.47 (0.81-2.66)	.203	**1.87 (1.02-3.43)**	**.043**
South England	8.27	1.43 (0.78-2.60)	.246	1.62 (0.88-3.00)	.121
Wales/Scotland	12.12	**2.18 (1.14-4.16)**	**.018**	**2.61 (1.35-5.03)**	**.004**

Social grade					
ABC1 (ref)	8.92	1.00		1.00	
C2DE	8.05	1.81 (1.10-2.97)	.539	0.81 (0.57-1.17)	.264

Family smoking					
None (ref)	7.33	1.00		1.00	
≥ 1 member	12.70	**1.84 (1.30-2.61)**	**.001**	**1.59 (1.05-2.42)**	**.030**

Family e-cigarette use					
None (ref)	7.89	1.00		1.00	
≥ 1 member	13.91	**1.89 (1.23-2.90)**	**.004**	1.42 (0.87-2.33)	.164

Number smoking friends					
None (ref)	8.15	1.00		1.00	
1	10.50	1.32 (0.83-2.12)	.246	0.86 (0.05-1.49)	.600
2	12.13	1.55 (0.80-3.03)	.195	0.72 (0.34-1.53)	.388
3	12.77	1.65 (0.74-3.69)	.224	0.60 (0.19-1.86)	.376
DK/Refused	7.25	0.88 (0.35-2.25)	.791	1.04 (0.37-2.96)	.938

Public approval of smoking					
Approve (ref)	16.66	1.00		1.00	
Disapprove	8.46	**0.46 (0.24-0.87)**	**.018**	0.69 (0.33-1.46)	.332
Neither	8.39	**0.46 (0.22-0.96)**	**.040**	0.62 (0.27-1.44)	.266
DK	5.70	**0.30 (0.11-0.85)**	**.024**	1.42 (0.45-4.42)	.548

Public approval of e-cigarettes					
Disapprove (ref)	7.91	1.00		1.00	
Approve	14.93	**2.04 (1.31-3.19)**	**.002**	1.44 (0.89-2.33)	.134
Neither	7.67	0.97 (0.65-1.43)	.867	0.85 (0.56-1.30)	.458
DK	3.77	0.46 (0.20-1.05)	.065	**0.40 (0.19-0.82)**	**.013**

Are e-cigarettes more or less harmful than smoking?					
Otherwise (ref)	4.49	1.00		1.00	
Less harmful	11.05	**2.65 (1.69- 4.14)**	**<.001**	**2.12 (1.32-3.41)**	**.002**
Constant				**0.02 (0.01-0.07)**	**.000**

DK = Don’t Know. ^1^% are weighted, N are the same as in Table 1. ^2^Analyses are adjusted for all variables listed.

## 4 Discussion

In this nationally representative sample of young people in Great Britain, as previously reported (Eastwood et al., 2017), just under two thirds (63%) have accurate harm perceptions of e-cigarettes relative to cigarettes. We also found that very few (9%) accurately perceive that nicotine causes little of the health harms of smoking. Accurate relative harm perceptions of e-cigarettes were higher among those who were older, had tried or used an e-cigarette sometimes, had at least one family member who used e-cigarettes, had no friends who smoke, perceived that the public approve or neither approves nor disapproves of e-cigarettes, and perceived that the public disapproves of smoking. Accurate harm perceptions of nicotine were higher among those who were older, had a family member who smokes, or who were unsure about whether the public approves of e-cigarettes. E-cigarette use was not associated with accurate harm perceptions of nicotine, and smoking was not associated with either harm perception. Accurate harm perceptions of e-cigarettes and nicotine were positively associated with one another, and this association was robust against all covariates included in this study.

The finding that the majority of young people accurately perceive e-cigarettes as less harmful than cigarettes, yet many still have inaccurate harm perceptions, is similar to some other studies of adults in Great Britain (Brose et al., 2015; Yong et al., 2017) and the US (Richardson et al., 2014). Harm perceptions of e-cigarettes relative to cigarettes have been reported from this dataset previously (Eastwood et al., 2017). However, the sample differed slightly, as the Eastwood et al. (2017) study involved cross-sectional data collected annually between 2013 and 2016 with respondents taking part in multiple waves and randomly assigned to one wave; the present study used all respondents from the 2016 survey. The Eastwood study also did not explore the prevalence of accurate harm perceptions of nicotine or correlates of accurate harm perceptions of e-cigarettes relative to cigarettes and nicotine.

The low prevalence rates of accurately perceiving that none or a very small amount, or even some but under half, of the harm from smoking comes from nicotine is a novel finding. A previous study among adult daily smokers from Sweden and Norway found higher rates of perceiving that none or a very small part (15%) or a relatively small part (31%) of the health risks from smoking come from nicotine (Wikmans and Ramström, 2010), yet these differences could be attributable to age, smoking status, or the availability of Snus in Sweden. Other previous studies in adults (Black et al., 2012; Borland et al., 2011) and US freshmen (Smith et al., 2007) have also found higher rates of perceiving that NRT is not harmful or less harmful than smoking (40%–80%); however, these differences could be attributable to the measures used. Black et al. (2012) assessed the harmfulness of long-term NRT use, Borland et al. (2011) of NRT relative to cigarettes, and Smith et al. (2007) of three specific NRT products relative to cigarettes. Further, as we found that accurate harm perceptions of nicotine were higher in older respondents, these differences may also be attributable to age.

The higher odds of having accurate e-cigarette harm perceptions in young people who used e-cigarettes less than monthly (vs. never users) is unsurprising and consistent with previous studies in adults (Adkison et al., 2013; Brose et al., 2015; Majeed et al., 2017; Yong et al., 2017) and young people (Ambrose et al., 2014; Amrock et al., 2015; Persoskie et al., 2017; Thrasher et al., 2016). However, there was no difference in harm perceptions among those who used e-cigarettes at least monthly (vs. never users); this is possibly due to less than 2% of respondents using at least monthly, which is reflected by the relatively wide confidence intervals for this group. The low prevalence of regular e-cigarette use is consistent with previous findings among young people in Great Britain (Bauld et al., 2017; McNeill et al., 2018).

Unlike e-cigarette harm perceptions, there was no association between accurate nicotine harm perceptions and e-cigarette use. Therefore, young people may be experimenting with e-cigarettes for reasons unrelated to harm perceptions of nicotine. This could be because not all e-cigarettes contain nicotine or because some individuals do not fully understand the role of nicotine in some e-cigarette use. However, harm perceptions of e-cigarettes and nicotine were positively associated with one another. Qualitative research and surveys including questions relating to these issues and the nicotine content in e-cigarettes may advance understanding of these findings.

Neither harm perceptions of e-cigarettes nor nicotine were associated with smoking status. This is contrary to previous studies in youth (Ambrose et al., 2014) and adults (Majeed et al., 2017), although there were several differences between those previous studies and the present study, including but not limited to different countries (US vs. UK), years of data collection, and covariates adjusted for. Longitudinal studies are necessary to assess whether accurate (or inaccurate) harm perceptions of e-cigarettes and nicotine are predictive of smoking uptake and other transitions, especially given recent debates surrounding the impact of e-cigarettes on youth smoking (Aveyard et al., 2018).

In terms of the demographic correlates, respondents who were younger had higher odds of being misinformed about harm perceptions of nicotine and e-cigarettes consistent with some previous studies (Ambrose et al., 2014; Amrock et al., 2015). There was little evidence for differences in social grade, which reflects findings in adults that there was no association between income and harm perceptions (Brose et al., 2015; Yong et al., 2017).

In terms of the social correlates, family use of e-cigarettes was associated with accurate harm perceptions of e-cigarettes. It is possible that some family members who use e-cigarettes were former smokers who overtly acknowledge the relative safety of e-cigarettes and benefits of switching. However, it may suggest that family e-cigarette use could increase the likelihood of youth e-cigarette use if perceptions translate to use, which would be of concern if youth were not smokers. On the other hand, exposure to family smoking was associated with accurate harm perceptions of nicotine (but not e-cigarettes). Number of smoking friends was not associated with harm perceptions of e-cigarettes or nicotine except responding “don’t know” or refusing to answer, which was associated with less accurate harm perceptions of e-cigarettes. Future studies could explore whether friends’ e-cigarette use influences harm perceptions.

This was the first study to explore the associations between public approval of e-cigarettes and smoking and harm perceptions of e-cigarettes and nicotine. Perceiving that the public approved of e-cigarettes yet disapproved of smoking was associated with accurate harm perceptions of e-cigarettes. However, public approval of these products was not associated with nicotine harm perceptions except responding “don’t know” or refusing to answer.

While this study provides important insight into the harm perceptions of e-cigarettes and nicotine in young people and variables associated with them, the findings must be considered in light of several limitations. First, data are cross-sectional, and therefore it is not possible to infer causality regarding the associations between harm perceptions and use of e-cigarettes/nicotine and product use. Specifically, it cannot be inferred whether young people with accurate harm perceptions of e-cigarettes and nicotine will subsequently try or be tempted to try e-cigarettes, tobacco cigarettes, and other nicotine products. Second, it is not possible to say whether these results generalise to other countries, and the generalisability of the findings to Great Britain may be limited as 10% of the sample was excluded due to having not heard of e-cigarettes or having missing data on key variables. Third, the data are from one time point in March-April 2016, and therefore caution must be exercised if attempting to generalise these findings over time, as harm perceptions are likely to be changing with the continued emergence of research, reports and media stories, and implementation of new laws and regulations (Brose et al., 2015). Fourth, consistent with other findings in Great Britain (Bauld et al., 2017; McNeill et al., 2018), rates of regular smoking and regular e-cigarette use were low, which may result in low power when drawing comparisons between these behaviours and harm perceptions. Low numbers of smokers and e-cigarette users also did not allow for exploration of dual use, which may be differentially associated with harm perceptions compared with exclusive use of either product. Fifth, the measure of nicotine harm perceptions could have been misinterpreted. While most of the health harms of smoking are caused by tobacco constituents other than nicotine, nicotine sustains smoking addiction (Benowitz, 2009) and may therefore be seen to increase exposure to toxicants. Indeed, Black et al. (2012) found that the most commonly reported harm of NRT was addiction. Future studies among young people should consider specific questions pertaining to the harm perceptions of nicotine in causing specific diseases such as cancers (as in Borland et al., 2011) and distinguish those from concerns about addiction and addiction promoting sustained tobacco use.

Despite these limitations, this study has important strengths. It is the first to assess prevalence rates of the harm perception of nicotine and the first to explore correlates of harm perceptions of e-cigarettes and nicotine among young people in Great Britain. Further, the sample was drawn from the general population in Great Britain using a quota sampling approach and subsequently weighted to enhance representativeness.

In conclusion, many young people in Great Britain have inaccurate harm perceptions of e-cigarettes and nicotine. E-cigarette use was associated with accurate harm perceptions of e-cigarettes but not nicotine, while smoking was not associated with either harm perception. Greater understanding of how these harm perceptions influence use of and transitions between e-cigarettes, tobacco cigarettes, and other nicotine products among both smokers and never smokers is needed. While some demographic groups may be more vulnerable to misinformation, specifically younger individuals, continued surveillance of harm perception in addition to nuanced information about e-cigarettes, nicotine, and smoking is essential for all.

## Contributors

HC and DA designed the survey, with input from AM, LB and SH. KE led the data analysis and write-up of the manuscript, with significant input from SH, LB and AM. All authors contributed to revisions of the manuscript and approved its final version.

## Conflict of interest

All authors declare no conflicts of interest.

## Role of the funding source

This study was led by Action on Smoking and Health (ASH) and was funded by the British Heart Foundation and Cancer Research UK core-funding of ASH. This study was conducted by YouGov PLC. KE, LB, SH, and AM are members of the UK Centre for Tobacco and Alcohol Studies (UKCTAS), which receives funding from The British Heart Foundation, Cancer Research UK, Economic and Social Research Council, Medical Research Council, and the Department of Health under the auspices of the UK Clinical Research Collaboration (MR/K023195/1). KE’s PhD is also funded by UKCTAS. LB is funded by a Cancer Research UK/BUPA Foundation Cancer Prevention Fellowship (C52999/A19748). HC and DA are employed by ASH which receives funding from Cancer Research UK, British Heart Foundation, and the Department of Health. Thanks are given to the UK Public Health Research Consortium (PHPEHF50/13) for funding the development of some of the covariates included in this study.
